# End-Expiratory Occlusion Test During Increase of Vasomotor Tone in a Rabbit Model of Hemorrhage

**DOI:** 10.1038/s41598-020-58096-2

**Published:** 2020-01-27

**Authors:** Juan P. Bouchacourt, Juan C. Grignola

**Affiliations:** 1 Department of Anesthesia, Hospital de Clínicas, Facultad de Medicina, Universidad de la República, Montevideo, Uruguay; 2 Department of Pathophysiology, Hospital de Clínicas, Facultad de Medicina, Universidad de la República, Montevideo, Uruguay

**Keywords:** Cardiology, Cardiology, Health care, Health care

## Abstract

End-expiratory occlusion test (EEOT) has been proposed as a preload responsiveness test that overcomes several limitations of pulse pressure (PPV) and stroke volume (SVV) variations. We compared the ability of EEOT versus SVV and PPV to predict fluid responsiveness during the increase of the vasomotor tone in a rabbit model of hemorrhage. Ten rabbits were anesthetized, paralyzed, and mechanically ventilated during basal load (BL), after progressive blood withdrawal (BW), and after volume replacement. Other two sets of data were obtained during vasomotor increase by phenylephrine (PHE) infusion in BL and BW. We estimated the change of stroke volume (∆SV_EEOT_) and aortic flow (∆AoF_EEOT_) during the EEOT. PPV and SVV were obtained by the variation of beat-to-beat PP and SV, respectively. Baseline PPV, SVV, ∆SV_EEOT_, and ∆AoF_EEOT_ increased significantly after BW, with a decrease of aortic flow (*P* < 0.05). PHE induced a significant decrease of PPV and SVV, but without affecting ∆SV_EEOT_, and ∆AoF_EEOT_. We conclude that ∆SV and ∆AoF during EEOT kept the ability to predict fluid responsiveness during PHE infusion in a rabbit hemorrhage model. This result may suggest the advantage of EEOT with respect to SVV and PPV in predicting fluid responsiveness during vasomotor tone increase.

## Introduction

The main goal of fluid therapy is to correct hypovolemia avoiding both under and over-resuscitation, as well as the excessive use of inotropic and vasopressor drugs. Moreover, the comorbidities and type of disease determine that not all patients are volume responsive. The preload responsiveness should be assessed to decide both to administer fluid in the early resuscitation phase of shock states and to stop fluid administration in the de-escalation phase of shock management^1^. Several indices are available at the bedside to predict fluid responsiveness. Dynamic parameters based on heart-lung interactions have been proposed to predict fluid responsiveness and to promote more rational fluid administration^2^. Nevertheless, respiratory variations of stroke volume (SVV), pulse pressure (PPV), and vena cava diameter have numerous limitations that reduce their accuracy and applicability in clinical scenarios^3^. By interrupting cyclic ventilation at end-expiration for a few seconds (end-expiratory occlusion, EEO), abolishing the inspiratory increase in intrathoracic pressure, and by passive leg raising tests, we can produce a self-volume challenge and increase cardiac output (CO) by a rapid and reversible increase of venous return (VR) in those patients preload-responsive^4,5^. Both tests have shown an excellent predictive value keeping their accuracy in patients with cardiac arrhythmias, with spontaneous breathing activity, with low compliance of the respiratory system and independently of positive end-expiratory pressure in acute respiratory distress syndrome patients^6,7^. However, passive leg raising presents some false negatives in patients with intra-abdominal pressure ≥16 mmHg, is contra-indicated to perform in patients with head trauma and intracranial hypertension and may be influenced by the use of compression stocking^4,8,9^. Further, the EEO technique does not have the technical constraints of passive leg raising, such as the measurements of CO by a fast and direct technique^10^.

In an animal model of hemorrhage, we have shown that the infusion of phenylephrine (PHE) (a pure α1-receptor agonist) blunts the dynamic preload indices increase after bleeding. This effect was mainly due to an increase in vasomotor tone^11^. Comparing the CO during and before the expiratory hold could be advantageous for the EEO test over SVV and PPV, maintaining the ability to predict fluid responsiveness beyond the vasomotor tone. The study aimed to examine the ability of the EEO test to predict fluid responsiveness under the increased vasomotor tone in a rabbit model of hemorrhage and to compare it with SVV and PPV.

## Results

### Hemodynamics

Table 1 shows the changes in hemodynamic data during different experimental conditions.

**Table 1:** Hemodynamic data during basal load (BL), basal load with phenylephrine infusion (BL + PHE), blood withdrawal (BW), blood withdrawal with phenylephrine infusion (BW + PHE) and volume replacement (VOL).

	BL	BL + PHE	BW	BW + PHE	VOL
AoF (mL/min)	71 ± 10	60 ± 7	50 ± 6*^,^**	47 ± 10*^,^**	75 ± 18
HR (bpm)	197 ± 30	186 ± 26	216 (29)	211 ± 30	211 ± 35
SV (mL)	0.34 ± 0.05	0.30 ± 0.09	0.22 ± 0.05*^,^**	0.22 ± 0.07*^,^**	0.32 ± 0.08
IVCF (mL/min)	66 ± 23	56 ± 17	49 ± 11	47 ± 9	66 ± 12
mPAo (mmHg)	71 ± 9	77 ± 11	71 ± 12	75 ± 13	73 ± 12
pPAo (mmHg)	35 ± 10	39 ± 12	38 ± 14	33 ± 16	34 ± 16
CVP (mmHg)	3.3 ± 2.0	4.6 ± 2.9***	0.7 ± 3.0	1.7 ± 3.0	4.8 ± 2.8***

*p < 0.05 vs. BL, **p < 0.05 vs. VOL, ***p < 0.05 vs. BW. AoF = aortic flow, CVP = central venous pressure, IVCF = inferior vena cava flow, mPAo and pPAo = mean and pulse aortic pressure, SV = stroke volume.

Hemorrhage (median blood volume withdrawal, 37 ± 6 mL, ≈14 mL**/**kg) induced a decrease of central venous pressure, inferior vena cava (IVC) flow, stroke volume (SV), and aortic flow, although only in SV and aortic flow reached statistical significance. Meanwhile, systemic arterial pressure (mean and pulse pressure) did not show a significant change.

PHE infusion induced a significant increase in vasomotor tone, evidenced by an increase in Ea_dyn_ from 1.1 ± 0.3 to 1.5 ± 0.7 (*P* < 0.05). During basal load (BL, euvolemia), PHE increased central venous pressure (*P* < 0.05) with a concomitant decrease of aortic flow and IVC flow. After blood withdrawal (BW, hypovolemia), PHE kept low values of the aortic flow, SV, and central venous pressure (*P* < 0.05) (Table 1). Mean and pulse arterial pressure, and heart rate did not show significant changes. Mean doses of PHE infusion was 14 ± 3 mcg/kg/min.

### End-expiratory occlusion test and dynamic preload indices

**Figure 1:**
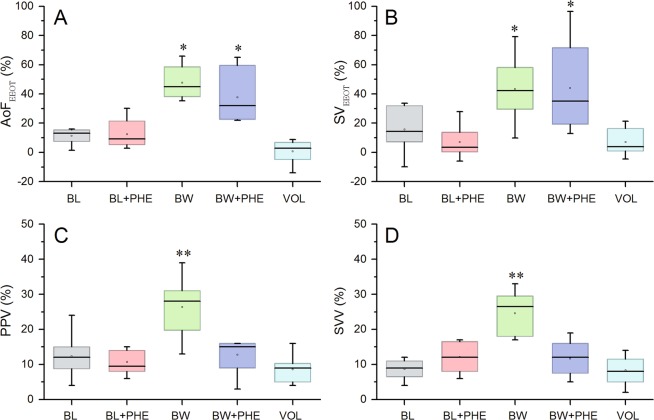
(**A**) Percentage of change of aortic flow before and during the end-expiratory hold (AoF_EEOT_), (**B**) Percentage of change of stroke volume before and during the end-expiratory hold (SV_EEOT_), (**C**) Pulse pressure variation (PPV), and (**D**) Stroke volume variation (SVV) during basal load (BL), basal load + phenylephrine (BL + PHE), blood withdrawal (BW), blood withdrawal + phenylephrine (BW + PHE) and volume replacement (VOL). The results are given by box representing the 25th to 75th percentile with lines indicating median and the error bars above and below each box represent the 90th and 10th percentiles, respectively. *p < 0.05 vs. BL, BL + PHE, VOL, **p < 0.05 vs. BL, BL + PHE, BW + PHE, VOL, using repeated measures ANOVA analysis.

**Figure 2:**
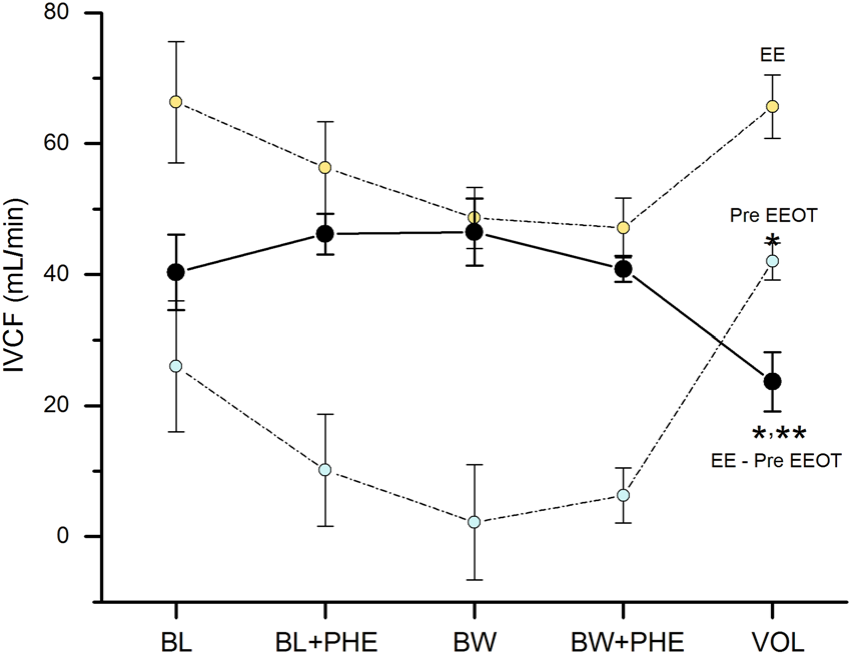
Absolute values of inferior vena cava flow (IVCF) during the end of expiration (EE) and the mechanical inspiration previous the end-expiratory occlusion test (Pre EEOT) and the difference between both (EE - Pre EEOT), during the different experimental conditions. Basal load (BL), basal load + phenylephrine (BL + PHE), blood withdrawal (BW), blood withdrawal + phenylephrine (BW + PHE), and volume replacement (VOL). The results are given as mean with error bars for SEM. *p < 0.05 vs. BW, **p < 0.05 vs. BL + PHE, using repeated measures ANOVA analysis.

BW increased SVV, PPV, ∆SV_EEOT_, and ∆AoF_EEOT_ (*P* < 0.05). However, after hemorrhage, PHE infusion decreased SVV and PPV (returning to BL values), without affecting ∆SV_EEOT_ and ∆AoF_EEOT_ (Fig. 1). Although IVC flow decreased after hemorrhage and during PHE infusion, its absolute change during the EEO test was preserved (Fig. 2).

During the EEO, the maximal aortic flow and SV were achieved at about 6 ± 3 sec.

## Discussion

The response of SV and aortic flow during the EEO test kept the ability to predict fluid responsiveness during PHE infusion in a rabbit hemorrhage model. This result may suggest the advantage of the EEO test with respect to SVV and PPV in predicting fluid responsiveness during vasomotor tone increase.

It is well known that fluid responsiveness is better predicted by the respiratory variation of SV and its pressure surrogates than by static measures of preload^12^. The EEO test has recently been proposed as a test for predicting preload dependence at the bedside that overcomes several limitations of SVV and PPV^6^, and it remains valid independently of the positive end-expiratory pressure in acute respiratory distress syndrome patients and even if the compliance of the lung is low^13,14^. In the present study, we add another advantage of the EEO test, since it holds the ability to predict fluid responsiveness in case of vasomotor tone increase during vasopressor drug infusion.

We must recognize that the mechanisms involved in the variation of the SV during mechanical ventilation and the EEO test are not the same. Mechanical ventilation induces cyclic changes in blood flow in vena cava, pulmonary artery, and aorta successively^15^. During a mechanical inspiration, the vena cava blood flow (i.e., VR and right ventricular preload) decreases first, and it has been related both to an increase in central venous pressure and to the compression of the vena cava due to the increase in pleural pressure. Concomitantly, right ventricular afterload increases secondary to the increase in transpulmonary pressure that opposes right ventricular ejection^16^. Both mechanisms result in a decrease in right ventricular SV and pulmonary artery blood flow that finally leads to a reduction in left ventricular filling and output, which is expressed during the expiratory period, a few heartbeats later^17^. Other two mechanisms that are expressed during the inspiration phase include the left ventricular preload increase due to squeezing the blood out of the capillaries toward the left ventricle (because of the rise in alveolar pressure) and the left ventricular afterload decrease due to the reduction of the systolic cardiac transmural pressure^18^. The EEO test consists of comparing the SV or CO during and before the expiratory hold. The interruption of the mechanical ventilation at end-expiration stops the cyclic increase of right ventricular impedance and the impediment in VR, which determines an increase in right ventricular SV that is observed at a systemic level (left ventricular SV, CO, and PP) in the following heartbeats. We cannot discard a concomitant left ventricle preload increase secondary to the maintenance of pulmonary capillaries opened inside the deflated alveoli by the decreased transpulmonary pressure during the end-expiratory hold^5^.

In hypovolemic conditions, the extent of the respiratory variation in SV is significant, mainly due to the expiratory decrease in left ventricular output that follows the higher inspiratory decrease in right ventricular output (often assessed as the “delta-down” of the systolic blood pressure) what determines an increase of both SVV (and its pressure surrogate, PPV) and the ∆SV_EEOT_ and ∆AoF_EEOT_^12^.

During the infusion of PHE, the rise of vasomotor tone would eliminate the expiratory component of the SVV or PPV (secondary to the inspiratory decrease of the right ventricular SV) and, it could attenuate the inspiratory increase of left ventricular VR (by hindering left ventricular filling due to the rise of ventricular diastolic pressure). However, it would not prevent the increase of the right ventricular SV secondary to the absence of the rise of transpulmonary pressure corresponding to the inspiratory period, keeping ∆SV_EEOT_ and ∆AoF_EEOT_ high in BW condition. Although BW and PHE decrease the VR, its absolute change was preserved, allowing to keep the predictive value of the EEO test.

The effects of vasoactive drugs on hemodynamics and especially on VR/CO interactions depend on many factors, including the plasma concentrations of the drugs, the relative density of α1, α2, β1, β2 adrenoreceptors and affinity for the receptor subtype^18^. Hence, one drug with even affecting only one type of adrenoreceptors can induce hemodynamic changes hard to predict. α1-adrenoreceptor agonists (such as PHE) constrict the arteries in systemic circulation, leading to a decrease in blood flow through the pre-capillary vessels and thereby decreasing the VR, and constrict the arteries of the splanchnic vasculature reducing the splanchnic arterial flow (leading to veins elastic recoil by a passive mechanism) and venoconstriction of vena cava and peripheral large and medium-sized veins (active mechanism) that work in concert to shift splanchnic blood volume to the systemic circulation increasing VR^19,20^. Approximately two-thirds of the increase in blood pressure during PHE administration resulted from an increase in VR with only one-third resulting from an increase in arterial tone. However, the concomitant increases in intrahepatic venous resistance can impede this blood volume shift, leading to blood sequestration within the splanchnic vascular bed and relative systemic hypovolemia^18^. We showed that PHE decreased both aortic and IVC flows, increasing mean and pulse aortic pressure (although without statistical significance) in BL condition. After BW, the decrease of aortic and IVC flows could not be modified by PHE. So, we can assume that the main effects of PHE administration in our model and the dose used are the increase of vasomotor tone ruling out any increase of VR, probably by a prevailing constriction of hepatic veins and vasculature within the liver.

At steady state, VR (IVC flow) and CO (aortic flow) are equal. According to Guyton, VR is defined by three variables: the mean circulatory filling pressure (MCFP), the right atrial pressure, which is clinically measured as the central venous pressure (CVP), and the resistance of venous return (Rv)^21^. The difference between MCFP and CVP is the pressure gradient for VR and is related to Rv by the following formula:

$${\rm{VR}}=\frac{{\rm{MCFP}}-{\rm{CVP}}}{{\rm{Rv}}}$$

MCFP is the pressure measured throughout the circulatory system when there is no blood motion and depends on the venous compliance and the intravascular stressed volume (volume of blood within the venous system under positive transmural pressure), which in turn depends on the total intravascular volume. Rv is a complex component that depends on the different parts of the venous circulation^19,22^.

**Figure 3:**
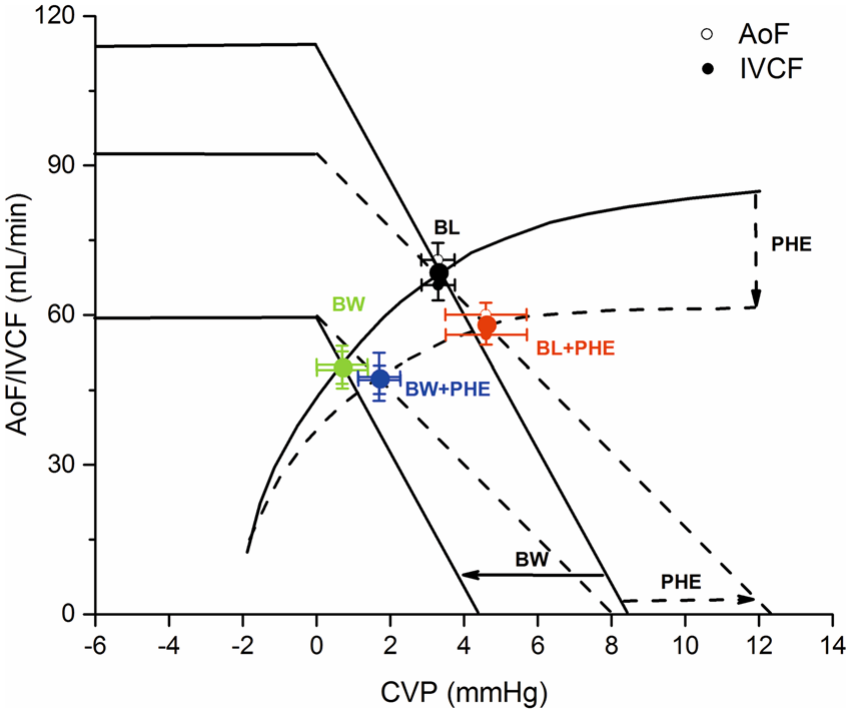
Possible effects of blood withdrawal (BW) and phenylephrine infusion (PHE) on inferior vena cava flow (IVCF) and aortic flow (AoF) curves as from the experimental data. BW would determine a parallel leftward shift of the venous return curve by a decrease of mean circulatory filling pressure (decreasing total and stressed volume without changes in the AoF curve, *solid lines*). PHE would determine an increase in ventricular afterload, shifting the AoF curve downward and a concomitant increase in venous return resistance (vasoconstriction of large veins and the vena cava) and mean circulatory filling pressure (vasoconstriction of venules and small veins, increasing the relative proportion of stressed volume to unstressed volume) (*dash lines*). CVP: central venous pressure.

Moderate hemorrhage (BW) determined a decrease of IVC flow with a parallel leftward shift of the venous return curve by lowering total and stressed volume without a change in unstressed volume resulting in a decrease in MCFP without significantly changing the Rv (Fig. 3)^18,23,24^.

As a direct α1-adrenergic receptor agonist, the net effect of PHE on the VR is determined by how much-stressed volume is recruited versus how much the downstream Rv increased^18^. In an experimental hemorrhage model, Cannesson *et al*. observed that in anaesthetized hypovolemic pigs, PHE boluses (from 0.5 to 4 mcg/kg) induces a dose-related increase in cardiac output secondary to an increase of IVC flow mediated by an unloading of the splanchnic reservoir and vice-versa, when it is preload independent, PHE boluses induce a decrease in CO associated with a decrease of IVC flow^25^. However, in a rabbit hemorrhage model, we have shown that PHE infusion (15 mcg/kg/min) induced a decrease in aortic flow with a significant SV decrease after BW compared with normovolemia. The different hemodynamic response to PHE may be probably related to the different doses of the α-adrenergic agonist (small boluses vs. relatively large continuous doses)^11,20^. We propose that PHE infusion had a predominant effect on the Rv (decreased slope of the VR curve without a change of MCFP), which tends to decrease VR. However, the concomitant increase in the relative proportion of stressed volume to unstressed volume, tend to offset some of the decrease in VR (shifting the VR intercept with the abscissa (MCFP) to the right). Finally, PHE infusion increases the left ventricular afterload, shifting the ventricular function curve downward (Fig. 3)^24,26^.

The current study has several limitations. Pulmonary hypertension and right ventricular dysfunction can mask hypovolemia and normalize the values of dynamic preload indices^27^. We discarded a significant increase in pulmonary pressure by PHE administration since ∆SV_EEOT_ and ∆AoF_EEOT_ remained elevated after BW. We analyzed the EEO test during PHE infusion in an animal hemorrhage model, which is not generalizable since the effects of vasoactive drugs depend on many factors including the plasma concentrations of the drug, the relative density of the subtypes adrenoceptors, the affinity of different catecholamines for the receptor subtype, and the cardiovascular function (i.e., vascular tone, intravascular volume status, myocardial contractility) at the time of administration^18^.

## Conclusions

We show that ∆SV_EEOT_ and ∆AoF_EEOT_ keep the ability to predict fluid responsiveness during PHE infusion in a rabbit model of hemorrhage. This result may suggest the advantage of the EEO test in predicting fluid responsiveness during hypovolemia and vasopressor drug administration.

## Materials and Methods

### Ethical considerations

This study was approved by the Ethics Committee on the Use of Animals (CEUA), Facultad de Medicina, Universidad de la República, Montevideo-Uruguay in October 2013 (Ethical Committee N° 070153-000363-13). We strictly complied with the Guide for the Care and Use of Laboratory Animals (NIH Publication N° 85-23, revised 1996), prepared by the National Academy of Sciences’ Institute for Laboratory Animal Research.

### Animal instrumentation

Ten female New Zealand rabbits (body weight 3.1 ± 0.6 kg) were premedicated with acepromazine (0.3 mg/kg i.m.) and meperidine (10 mg/kg i.m.). We placed a fluid-filled catheter (24-gauge) through an ear vein to induced anesthesia (midazolam 0.5 mg/kg i.v.) and to administrate saline solution (7 mL/kg/h) as maintenance requirements^28^. The animals were tracheotomized and mechanically ventilated (Amadeus Hamilton Medical AG, Switzerland) via an endotracheal tube (ID 2.5 mm). The ventilator was set in the volume-controlled ventilation mode with a tidal volume of 8 mL/kg, end-expiratory pressure of 5 cmH_2_O, a respiratory rate 38 ± 6 breaths/min, and inspired fractional oxygen of 60%. Peripheral oxygen saturation (Rainbow 7 LNOP newborn sensor) was monitored and maintained above 96% (Radical 7 pulse oximetry monitor, Masimo Corporation, Irvine, CA, USA). The respiratory rate was adjusted to maintain an end-tidal CO_2_ tension between 30–38 mmHg (Datex Inst Corp CD-200-43-00, Helsinki, Finland). The arterial partial pressure of oxygen and carbon dioxide were monitored by serial blood gas evaluation (ABL520, Radiometer, Denmark). The heart rate/respiratory rate ratio was >3.6. Anesthesia was maintained with a continuous infusion of midazolam (0.5–1 mg/kg/h) and rocuronium bromide (0.6 mg/kg/h). We used a heating pad to keep the animal normothermia.

We placed a 4.5 F triple-lumen central venous catheter (Paediatric Multicath 3-Vygon) in the left jugular vein for measuring central venous pressure, blood withdrawal, and drug infusion. After making a right lumbar incision (extra-peritoneum approach), two non-constricting ultrasonic perivascular flow probe (2.5PSB-Series Flow probe, Transonic Systems Inc., Ithaca, NY, USA) were positioned one around the infra-diaphragmatic aorta and the other around the inferior vena cava. Both sensors were connected to a Doppler flowmeter (model T201, Transonic Systems Inc., Ithaca, NY, USA) to measure instantaneous aortic and IVC flow, respectively. We monitored systemic arterial blood pressure placing a fluid-filled catheter (20-gauge) through the right femoral artery up to the infra-diaphragmatic aorta, just distal to the aorta flow probe. All pressure transducers (P23Db Gould Statham) were zeroed to atmospheric pressure at the midthoracic level.

### Experimental protocol

Once the instrumentation was completed, the animals were allowed to stabilize for 30 min, and all the variables were obtained during basal load (BL). After BL measurement, a second set of hemodynamic data were obtained after increasing the vasomotor tone by PHE infusion (Sigma, St. Louis, MO) during 30 min (BL + PHE). Twenty minutes after stopping PHE infusion, we removed blood progressively by stepwise cumulative volume of 5 mL/kg with a total of 15 mL/kg of body weight (15% of volemia) and a third set of hemodynamic measurements was obtained (BW). After thirty minutes of blood withdrawal, a fourth set of data was obtained during PHE administration (BW + PHE), and finally, a fifth set of data was obtained after volume replacement (VOL, 14 mL/kg) with HAES-6% (Fresenius Kabi, Deutschland GmbH, Germany). We used the PHE to increase the vasomotor tone^11^.

Once we completed the experimental protocol, the animals were killed with intravenous potassium chloride under deep anesthesia.

### Data collection and analysis

We monitored all signals in real-time and stored digitally at the end of expiration (SAMAY M16, sampling frequency of 200 Hz). Hemodynamic values were averaged over a 5-sec period. SV was estimated offline from the integral of the systolic portion of the aortic flow curve for each cardiac cycle^11^.

**Figure 4:**
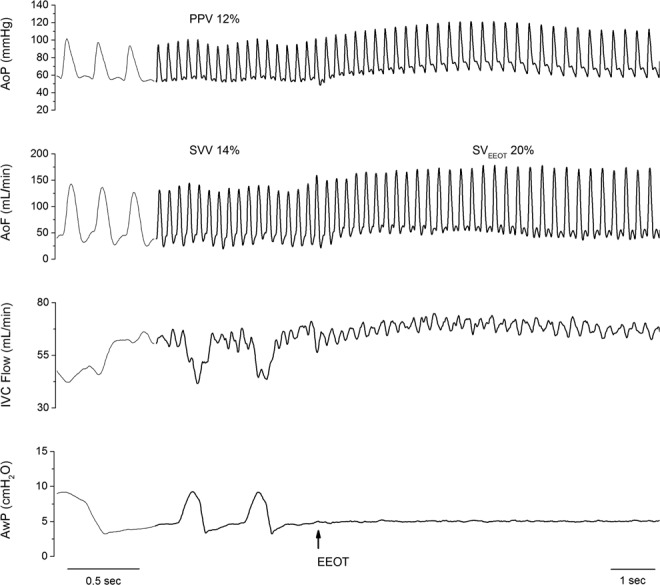
Representative traces of the different hemodynamic variables during mechanical ventilation and end-expiratory occlusion test (EEOT) in the baseline condition. AoF and AoP = aortic flow and pressure, respectively; IVC Flow = inferior vena cava flow; AwP = airway pressure; PPV and SVV = pulse pressure variation and stroke volume variation, respectively.

We performed one EEO test in each experimental condition by interrupting the ventilator at end-expiration over 10–15 sec (Fig. 4)^5^. We calculated the change of SV (∆SV_EEOT_), and aortic flow (∆AoF_EEOT_) from the value before the expiratory hold to the value corresponding with the maximal aortic flow reached during the EEO. The hemodynamic changes were expressed as percent changes from the value before the expiratory hold.

SVV and PPV were assessed using the standard formulae (100 × (Q_max_ − Q_min_)/[(Q_max_ + Q_min_)/2], where Q_max_ and Q_min_ are the maximum and minimum values for SV and PP during the same respiratory cycle, respectively^17^. We averaged the measurements of three consecutive respiratory cycles for statistical analysis.

The vasomotor tone was assessed by the dynamic arterial elastance (Ea_dyn_ = PPV/SVV)^29^.

### Statistics

Preliminary experiments were employed to calculate the sample size. A sample size of ten animals allows detecting a 20% effect in ∆SV_EEOT_ and ∆AoF_EEOT_ (standardized difference of 1.25: target difference/standard deviation) for a level of significance of 0.05 and a power of 80%^30^. Normal distribution was tested with the Shapiro-Wilk test. Data are expressed as mean ± SD, except other thing was stated. SVV, PPV, and EEO test were analyzed at the different experimental conditions using a one-way analysis of variance (ANOVA). Post-hoc testing was performed using the Bonferroni test. Paired Student’s t-test was used to compare the different values of Ea_dyn_ under PHE infusion (BL + PHE and BW + PHE) versus without PHE infusion (BL, BW, and VOL). All statistical tests were performed using SPSS software (Version 21.0; SPSS Inc., Chicago, IL). P-value < 0.05 was considered statistically significant.

## Data Availability

All relevant data supporting the conclusions of this article is included within the article.
